# The effectiveness of a sexual assault nurse examiner-grounding program (SANE-GP) on knowledge, skill and practice regarding sexual assault examination (SAE) among nurses working in a tertiary care hospital in Udupi district, India: A study protocol

**DOI:** 10.12688/f1000research.74978.2

**Published:** 2022-12-13

**Authors:** Renjulal Yesodharan, Vinod Nayak, Tessy Jose, Vikram Palimar, Anice George

**Affiliations:** 1 Department of Psychiatric Nursing, Manipal College of Nursing, Manipal Academy of Higher Education, Manipal, Karnataka, 576104, India; 2 Department of Forensic Medicine and Toxicology, Kasturba Medical College, Manipal, Manipal Academy of Higher Education, Manipal, Karnataka, 576104, India; 3 Department of Peadiatric Nursing, Manipal College of Nursing, Manipal Academy of Higher Education, Manipal, Karnataka, 576104, India

**Keywords:** Forensic nursing, Forensic Science, SANE, sexual assault, sexual violence

## Abstract

The medico-legal care of victims of sexual assault is very challenging, and requires specific knowledge and skills. Professionals in the emergency departments of hospitals might not have specialised training in forensic science. Nurses have a very significant role in these settings, but they lack any formal forensic training. This study aims to develop a sexual assault nurse examiner-grounding program (SANE-GP) for Indian nurses to inculcate knowledge and skill regarding sexual assault examination. The study adopts a three-stage Delphi technique to develop the training module and uses a time-series design to evaluate the effectiveness of the program. A questionnaire on nurses’ knowledge on sexual assault examination (KQSANE-I) will be developed in phase-I and subsequently used in phase-II. The protocol of SANE-GP will help the medical community to implement the program across India. The implementation of SANE-GP can also help to start a sexual assault nurse examiner network.

## List of abbreviations

ASO: Alternative Sexual Orientation

CrPC: Criminal Procedure Code

DAC: Dissertation Advisory Committee

KQSANE-I: Questionnaire on nurse’ knowledge about sexual assault examination

POCSO: The Protection of Children from Sexual Offences Act

SAE: Sexual Assault Examination

SAFE: Sexual Assault Forensic Examination

SANE: Sexual Assault Nurse Examiner

SANE-GP: Sexual Assault Nurse Examiner-Grounding Program

WHO: World Health Organization

## Introduction

Sexual violence is a huge concern and has malignantly affected all strata of our community regardless of age, gender, race, ability, and social status. However, the estimates of incidents of sexual violence around the globe suggest that there is a predominance of sexual violence against women in comparison to the sexual violence against men. The annual report “Crime in India – 2019” by the National Crime Record Bureau (NCRB) indicated that a total of 4,05,861 cases of crime against women were registered in India during 2019, which includes 7.9% of rape cases. The crime rate registered per lakh women population was 62.4 in 2019 in comparison with 58.8 in 2018 ( National Crime Records Bureau (NCRB), 2019). Sexual violence affects many aspects of the victim’s life including personal safety, their family, interpersonal relationships, finance, and work environment, and makes the victim undergo perplexity of investigation and legal trial. Unfortunately, in some cases the medical, legal and law enforcing agencies operating in the country and the society itself, make the victim feel the sexual assault was their fault and re-victimise them for someone else’s cruelty. ( Relyea and Ullman, 2017). Most of the time, the trauma they are already undergoing, is much less than the trauma they receive from the system — a system that is supposed to help them ( Maier, 2008). Sexual offenses are under-reported to law enforcement agencies due to fear of retaliation and humiliation ( Shanmugam, 2013; Flecha, 2021). A comparative study of the NCRB data on rape and the National Family Health Survey shows that only 0.6% of women who faced sexual violence filed a complaint to the police ( Rukmini, 2014). The legal system of India is common to all states and union territories. The judiciary is independent and not governed by the federal government. The time taken for a case to come to trial in the Indian court is getting longer. Crimes against women and an increase in the incidents of criminal activities have contributed to the workload of the judiciary system ( Jaithlia and Maheshwari, 2020).

When victims are being re-victimised by the system, most of them will not go to the hospital and report that they were sexually assaulted and are also not willing to follow the criminal justice system ( Maier, 2012). This causes a significant underestimation of the prevalence of crimes against women and children ( Murshid and Bowen, 2018; Jennings, Powers and Perez, 2021). The Criminal Law Amendment Act 2013 widened the definition of rape and acknowledges the right to get medical and forensic attention to all victims and survivors of sexual assault by health care centres in public as well as private sectors. Failure to provide health care services is now considered an offence under this law. The right to treatment requires the state to guarantee that adequate, good quality proper medical, forensic, and mental health services are accessible to the victims without any discrimination ( Ministry of Health and Family Welfare (MOHFW), 2014). An excellent health care service requires professionals who can treat injuries, do medico-legal examinations, collect evidence, provide prophylaxis, test for sexually transmitted infections, administer emergency contraception, and give psychosocial support to the victims.

The Indian health care delivery system is constituted in such a way that most of the reported sexual assault cases are managed by the emergency departments of the hospitals that do not have health care professionals with specialised training in forensic science ( Saxena *et al.*, 2015; Lingam *et al.*, 2021). The situation is similar in state-run as well as in private hospitals, except for certain medical colleges. The emergency team consists of doctors, nurses, and paramedics, in which doctors have the minimum qualification of Bachelor of Medicine and Bachelor of Surgery (MBBS). The curriculum of MBBS contains a para-clinical subject of ‘forensic medicine including toxicology’, which is a stand-alone exposure to the field of forensic sciences. The Indian legal system give preferences to female doctors for the examination and evidence collection from the survivors of sexual assault ( Ministry of Health and Family Welfare (MOHFW), 2014). However, the number of female doctors in the country are low ( Rao *et al.*, 2011) and female nurses are more in number and they are easily accessible to the system and well accepted by the survivors. When considering the cost of the basic health care services nurses are cost effective compare to the doctors. In any medical or health care setting nurses are the ‘first point of care’ and have very crucial role in the examination and evidence collection from the victims of sexual assault ( Yesodharan *et al.*, 2022e). The female nurses are sensitive to their patients’ physical and emotional needs and able to develop early rapport with the victims of sexual assault, which will lead to better acceptance and allows the victim to open up during the time of history collection and medico-legal evidence collection ( Campbell *et al.*, 2013). However, nurses lack forensic training in any form, and the casual examinations can cause missing of vital evidences ( Dash *et al.*, 2016).

In developed countries, nurses have a very significant role in the field of emergency and trauma care, toxicology, crime scene investigation, and correctional settings ( Henninger *et al.*, 2020). Strengthening forensic nursing education in the country is critical as the crimes against the women and children are increasing. There is a wider scope for forensic nursing in India and the nurses can take roles in medico legal investigations and evidence collection ( Lynch, 2011; Renjith *et al.*, 2016).

### Research gap

Medico-legal care of the victims of sexual assault is very challenging as it involves medical, legal, and ethical aspects. The curriculum of BSc Nursing, as well as General Nursing and Midwifery, does not deal with forensic clinical examination and the collection and preservation of evidence. Unlike the sexual assault referral centres (SARC) in the global scenario, India does not have such designated centres, and the victims are taken care of by the health care centres and state-run hospitals.

The nurse in the health care centres needs to be competent enough to handle the medico-legal cases that require specific knowledge and skills ( Alsaif *et al.*, 2014). Training on a wide range of issues is important for the nursing staff handling medico-legal cases ( Zerbo *et al.*, 2018). These include positioning and examination of the client, obtaining high-quality specimens, identifying and reporting genital-anal injuries after sexual assault, comparing the injuries of consensual and non-consensual intercourse, identifying and collecting traces from the fingernail and other materials for the DNA analysis of the aggressor and employing DNA evidence in sexual offence cases to aid the identification of suspects, movement of forensic evidence and chain of custody, analysing and preventing drug-facilitated crimes against women, usage of photo colposcopy to identify hymenal transections and other injuries in children and adult females, simulation, forensic photography, bite-mark identification, handling vicarious trauma and mindfulness based interventions for better coping. Peer support is also essential for nurses to be able to cope with the demands of working in such situations ( Drake and Adams, 2015; Gharedaghi *et al.*, 2018; Yesodharan *et al.*, 2018, 2021; Rodriguez *et al.*, 2019; Usman *et al.*, 2019; Yassa and Badea, 2019; McAllister and Vennum, 2021; Zweig *et al.*, 2021).

A lacuna is identified in the literature related to forensic examination of sexual assault victims. A few studies were identified which assess the knowledge of nurses and student nurses in forensic medical examination. However, no studies were reported from India or Asia regarding the knowledge and skills of nurses in forensic examination and evidence collection.

### Conceptual framework

The attribute service quality model by Haywood-Farmer was adopted as the conceptual framework for this study ( Haywood-Farmer, 1988). This model states that a service organisation is termed high quality if it consistently meets customer preferences and expectations. The separation of attributes into various groups is the first step towards developing service quality. The three types of quality attributes of health care services are physical and process components, behavioural elements, and elements of professional judgment.

### The study

The proposed research is designated to determine the level of the nurse’s competence and knowledge in the examination and management of sexual assault and investigate the effectiveness of multicomponent training in improving knowledge and skill. Implementation of multicomponent training enables nurses to communicate efficiently, examine precisely, collect and preserve evidence accurately and present it properly before the court of law.

The increase in the number of sexual crimes against women and children, stringent guidelines and protocols issued by the government, the influence of mass media, and increased social pressure demands health care professionals to be more accurate and sensitive in handling cases of sexual assault. The findings of the study will help to develop a protocol for nurses, and the same can be utilised as a model guideline for nurses in India. The findings of the study are expected to initiate the sexual assault nurse examiners network (SANE network) in the Udupi district, which can be adopted to other districts.

### Research objectives

i. Develop a valid and reliable instrument to measure the knowledge regarding SAE among nurses
ii. Develop a SANE-GP module using a three-stage Delphi method
iii. Explore the effectiveness of SANE-GP on the knowledge and skill regarding SAE.
iv. Develop a platform for the SANE network in the country

## Protocol

### Design

The proposed study adopts a cross-sectional survey design in Phase-I and an experimental design to meet the objectives in Phase-II. The study also uses a Delphi technique for the development of a module for the SANE-GP.

*Phase-1*

Objective 1: A questionnaire on nurses’ knowledge about sexual assault examination-(KQSANE-I) will be developed based on the Robert F DeVellis method of tool development ( DeVellis, 2016), which includes item development, item pooling, content adequacy assessment, clarification of the items, refining the items, the administration of questionnaire (empirical testing after determining the scale for items and adequate sample size), item analysis, reliability assessment, and construct validity. The administration of the questionnaire will be conducted through a survey, and the data will be collected from 450 participants. Skill questionnaires and practice checklists will be developed along with the development of the SANE-GP module.

Objective 2: Delphi techniques of data collection with experts in the field of forensic science to develop a SANE-GP module for nurses.

*Phase-2*

Objective 3: The study proposes to have one group with a before and after experimental design with three follow-ups to evaluate the effectiveness of SANE-GP among nurses working in the hospital.

### Research setting and population

Phase-I of the study will be carried out amongst the registered nurses from private and state-run hospitals from Udupi and Dakshina Kannada districts of Karnataka, India. Participants will be included from both genders. A three-stage ‘Delphi’ technique will also be initiated to develop the module for Phase-II. The experts are from the field of forensic medicine and forensic nursing from the national and international arena. In the second phase, 74 nurses from a selected tertiary care hospital will be recruited for the training of SANE-GP and subsequent assessment of the effectiveness of the program.

### The sampling technique

In Phase-I, the item pool, which is developed after reviewing the current literature and validated with the experts, will be administered to 450 registered nurses who are willing to participate (see *Extended data,* Yesodharan *et al*., 2022a). The sampling technique adopted is purposive sampling. For the development of the module for SANE-GP, Delphi technique data collection will be conducted in three stages. Each stage of Delphi will have seven experts from the field of forensic medicine and forensic nursing. A five-step process will be utilized for the selection of the panel members: i) review the literature and make a list of potential experts based on the recent work in sexual assault examination, ii) check citation index for number of citations, iii) evaluate each expert’ work and grade them on a scale of three, iv) present the potential experts’ work to the Dissertation Advisory Committee (DAC) and develop and final list of experts, v) contact each expert through mail or telephone and explain the purpose with an invitation to participate. A personalised email invitation will be sent to the experts agreed to participate in the study.

### Sample size

The sample size is calculated for Phase-I based on the item to response ratio of 1:10 given by Schwab (1980). The KQSANE-I is planned to have a minimum of 44 items, hence, the sample size calculated for Phase-I is 450. For Delphi, seven experts will be recruited for participating in the study. The sample size calculated for Phase-II is based on the time series analysis using the formula n = [Z _1-α/2_ + Z _1-β_] ^2^ σ ^2^ [1 + (m − 1)ρ]/md ^2^ (where, n is the sample size, Z _1-α/2_ at α = 0.05 = 1.96, Z _1-β_ at 80% power = 0.84, σ = standard deviation (23.75), ‘m’= number of follow ups (3), ‘ρ’ = intraclass correlation (0.4), ‘d’ = clinically significant difference (7)). The sample size is calculated with an anticipatory non-response rate of 25% which is 74. The participants will be recruited through purposive sampling for SANE-GP.

### Inclusion criteria

Study participants for Phase-I: Registered nurses working in selected hospitals will be included in the study. The hospitals will be selected based on the convenience of the researcher.

For Delphi technique: Five to seven experts in the field of forensic science and forensic nursing will be selected.

Study participant for Phase-II: Female registered nurses from 22 to 45 years working in a tertiary care hospital will be included in the study.

### Data collection

Phase-I: Delphi method to finalise the content of the SANE-GP training module. Permission from the respective hospital management will be obtained before the advertisement for the recruitment of nurses to the study. The study process will be informed in detail through an information sheet, and informed consent will be obtained from the participants during Phase-I. The researcher will be present throughout the data collection. Phase-I of the study also contains the development of a training module; a Delphi method will be used to finalise the content of the SANE-GP training module.

The researchers will do a literature search and prepare the initial draft of the module and the knowledgeable subject experts in DAC will assess the readability of the initial draft. Revisions will be made on the draft module based on their suggestions. When the expert panel list is finalized, a discussion forum will be setup on an online platform by keeping the details of the experts anonymous. The draft module and the other the questionnaire will be made available to the experts either through email or uploading to the online forum. A stage one of Delphi will be initiated and the experts will go through the draft module and assess based on questionnaire. The questionnaire involves items such as whether the content is worded correctly, whether the content is relevant, and whether the content is adequate. All the items have to be graded using a four-point Likert rating scale. The experts will have one-month time to the overall assessment of the draft module. When the responses (Scores and comments) from all the panel experts are available then it will be scrutinised by the researchers and identifies the common and conflicting viewpoints. The draft module will be revised based on the suggestions by the expert panel. The stage one of the Delphi ends with preparation for the stage two. In the stage two of Delphi the scores and the comments from the experts are mailed to the experts and the experts are encouraged to revise their earlier views in light of the replies of other members of the group. The experts will grade the draft module based on the items in the questionnaire and the disagreements will be resolved by discussing and negotiating with the experts. The draft module will be further revised and move forward for the final stage of Delphi. The process will be repeated until all the panellists in the Delphi reach a consensus. A thematic framework will be used for the analysis of the information collected through the Delphi method.

Phase-II: The effectiveness of the SANE-GP will be assessed through administering KQSANE-I. A pretest will be conducted on day one, and an immediate post-test will be conducted after the intervention. Follow-ups will be conducted on the 8th, 16th, and 24th week after SANE-GP. The skill of the participants in performing SAE will be assessed after the completion of each section of the module through a skill checklist. After the implementation of SANE-GP, the participants will be randomly picked for assisting the forensic expert whenever a sexual assault victim is coming to the hospital for examination. The implementation of the SANE-GP will be assessed by assessing the practice of the nurses by the forensic expert (outcome assessor) through a checklist.

### Data collection instruments

*Tool 1: Demographic Proforma*

Demographic proforma includes items such as age, gender, occupation, highest education, years of experience, specialty, and previous experience in SAE.

*Tool 2: KQSANE-I*

This will be used for assessing the knowledge of participants regarding the sexual assault examination. The tool will be developed in Phase-I after completing all tool development steps, namely, item development, item pooling, content adequacy assessment (clarification of the items and refining the items), the administration of the questionnaire (empirical testing after determining the scale for items and adequate sample size), item analysis, reliability assessment and assessing construct validity.

### SANE grounding program (SANE-GP)

The SANE-GP will have multiple teaching-learning activities given to the participants for a period of one week. Each session will last for 45 min and seven sessions will be conducted per day. The content of each module and the order of the sessions also will be finalized after the third stage of the Delphi. Additional modules will be also added based on the suggestions of the expert panel. The details of the modules which will be prepared and send to the panel experts are mentioned below.

1. **Overview of forensic and sexual assault examination**
  i. *History of medico-legal examination*
  ii. *The constitution of India and human rights*
  iii. *Criminal behaviour and criminal body language*
2. **Male, female, intersex and ambiguous genitalia**
3. **Medico-legal history taking**
  i. *Communication and soft skill development (for caring of victims)*
  ii. *Sexual abuse against children*
  iii. *Sexual violence against transgendered persons, intersex persons, and persons with alternative sexual orientations (ASO)*
  iv. *Sexual violence against elderly*
  v. *Prerequisites for history collection and examination*
4. **Observing and assessing physical examination findings**
  i. *Local examination of genital parts and other orifices*
5. **Medico-legal evidence collection**
  i. *Informed consent*
  ii. *Evidence and types of evidence*
  iii. *Crime scene investigation*
  iv. *Sexual assault forensic examination (SAFE) kit*
  v. *Body evidence and anogenital evidence collection*
  vi. *Collection and packing, and preservation of material evidence from the survivor*
  vii. *A chain of custody*
6. **Forensic photography, colposcopy, and other digital evidence**
  i. *Cameras and accessories*
7. **Medical management of cases with sexual violence**
  i. *Standard operating protocol*
8. **Sexually transmitted infection testing and prophylaxis**
9. **Pregnancy testing and prophylaxis**
10. **Medico-legal documentation**
  i. *Examination of injuries and intimation to police*
11. **Legal consideration and judicial proceedings**
  i. *The Criminal Law Amendment Act 2013*
  ii. *The Indian Evidence Act 1872*
  iii. *The CrPC and the Indian Penal Code*
  iv. *The Protection of Children from Sexual Offences Act, 2012 (POCSO Act)*
  v. *Legal responsibilities of health professionals*
12. **Community collaboration**
  i. *Dealing with police and judiciary and child welfare committee*
  ii. *Dealing with public and mass media*
13. **The psychosocial care of victims and family members**
14. **Networking of SANE nurses**

The SANE-GP also includes practical activities such as case discussions, discussion of supreme court and trial court verdicts, communication and soft skills demonstrations, documentation of injuries, mock case examinations, simulations, dummy examination, and preparation of slides with the specimen collected from the vaginal vault and other orifices using swabs.

### Ethical consideration

Institutional Ethics Committee of Kasturba Hospital and Kasturba Medical College Manipal, Karnataka, India, approved the proposed study through wide reference No 653/2018. Complete information about the study will be given to the participants through an information sheet and written informed consent will be obtained (see *Extended data,* Yesodharan *et al*., 2022b, c, d).

### Expected outcomes

The current study is aimed at assessing the knowledge, skill, and practice of nurses regarding sexual assault examination. The knowledge will be assessed before SANE-GP, and three follow-ups will be conducted on the 8th, 16th, and 24th week after SANE-GP. The researchers will meet the participants in their work area and administer the questionnaires physically. The skill will be assessed immediately after the completion of each module in the SANE-GP whereas the practice of the participants will be assessed randomly in the presence of the forensic expert from the research setting using a practice checklist. Once the participants complete the SANE-GP, they will be enrolled in an association registered under the Societies Registration Act, 1860. This platform will be used for the communication and future development of SANE programs in India.

### The plan for data analysis

The researchers will use statistical software by IBM (IBM SPSS Statistics, RRID:SCR_019096), SPSS statistics 26 (Armonk, NY: IBM Corp) for the analysis. In Phase-I, the data will be summarised using descriptive statistics and will be presented in a summary table. The knowledge score will be assessed four times (one before SANE-GP and three follow-ups), and the scores will be compared and analysed with the help of time-series analysis. The skill of the participants will be assessed after the completion of each module, and the score will be described in tables. The practice of the random participants will be assessed and described.

### Dissemination

Results will be disseminated via presentations at appropriate scientific conferences and meeting of professional bodies. The findings will also be published in peer-reviewed journals, professional and institutional repositories etc. The result will be discussed with the Governmental bodies and other stakeholders for improvement of process in medico-legal evidence collection.

### Study status

The study team is in the process of recruitment of participants for the phase I of the study.

## Discussion

Forensic examination of the sexually assaulted individual has multifaceted challenges ranging from therapeutic to legal. To take care of such individuals, the forensic team including the nurse should be competent enough to deal with the challenges emerging out. A forensic examination is not everybody’s business; keeping it open to all may hamper the evidence and affect the admissibility of the evidence in the court of law, helping the culprits to escape. An unprofessional and unskilled way of collecting evidence can make victims re-experience the trauma and lead to physiological and psychological distress. A study conducted by Lingam *et al.* (2021) reported that medical professionals did not gather several important evidence during the sexual assault examination which included the absence of proper sample collected, missing certain key procedures in medicolegal evidence collection and they gave more emphasis to physical examination rather than the symptoms of psychological consequences of an assault. The study also suggested training in sexual assault examination and evidence collection to the medical professionals. The study also concluded about the need for a holistic policy and program that directs health providers’ attention to the needs of survivors after an assault ( Kashyap & Gerntholtz, 2010).

In India, the SAE is currently done by a forensic expert or a clinician/physician with the assistance of a nurse. There are lot of shortcomings in this model. Lack of knowledge of doctors when comparing to the forensic medicine experts, shoddy service, lack of specific training ( Lingam *et al.*, 2021), gender biased practices such as two finger test, commenting on the past sexual history of the survivor ( Fatima *et al.*, 2018), violation of rights of the privacy of the survivors, reporting biased medical opinions such as “habituated to sexual intercourse” ( Bandewar *et al.*, 2018) improper reporting without consent of survivors ( Pitre & Lingam, 2022).

The nursing professional who is assisting is not trained or taught forensic evidence collection. Moreover, the communication with the legal authorities and the victim may be affected if the nurse is not trained to deal with those types of cases. The Indian medico-legal system only allows doctors to conduct the medico-legal examination, unlike the United States of America or the United Kingdom, which allows competent and licensed Sexual Assault Nurse Examiners (SANE) to do so. We do not have any specialised course or any accrediting agency to certify the nurses to do the sexual assault examination, which compromises the care of the victim, collection of evidence, and reporting of facts to the court.

The current research is important because for the first time in India, it proposes a minimum requirement for the training of nurses regarding sexual assault examination. The research also proposes a guideline for the practising of sexual assault examination by the nurses. The specific role of the trained nurse increases the accountability and responsibility in the evidence collection and care of the victims. The study also proposes a network of forensic nurses so that all cases reporting in any hospitals, nursing homes, and clinics will get the service of the trained nurse irrespective of their current employment.

A study by Cunha *et al.* (2016) evaluated the knowledge of nursing students over forensic practices using a KQFNP, which showed that there was an insufficient knowledge over the practical aspects of forensic nursing (36.3%). The study highlighted the importance of enhancing the training of the nursing students about the forensic nursing aspects allowing them to adopt good clinical practices.

Cucu *et al*. (2014) conducted a descriptive study in the transversal approach and identified and described the knowledge, experiences, and training needs of nurses related to forensic patients. In total, 30 nurses from the emergency department were assessed with a 53-item self-administered questionnaire. The results of the study based on correct answer scores showed that a small group of the participants (13%) performed poorly (less than 5), and a majority (63%) did relatively well (6-10) than the former on a 17-item knowledge questionnaire. The study results also revealed that all the nurses agreed (100%) that they required solid training on forensic topics. The participants also ranked ten specific topics areas concerning forensic patient care, and the major five were forensic patient communication (4.7 ± 0.88), the legal aspect of forensic medicine (4.67 ±0.80), violence (4.57 ±0.97), traffic accidents (4.53 ±0.86), and sexual assault (4.43 ±1.07). The survey concluded that forensic training is desirable and needed among emergency department nurses as an assurance to render appropriate care together with proper management of medico-legal evidence and advocate for the patient rights.

Feizi Nazarloo *et al*. (2017), through a cross-sectional study, revealed that emergency nurses had the least knowledge in the collection and protection of forensic evidence. Among the 195 participants, 95.4% had no formal training in managing the forensic patients, 92.3% had stated that there was no formal written guideline in caring for forensic patients, and 95.9% had educational needs for managing the forensic patients. The overall knowledge status about forensic nursing is low in emergency nurses (44.13%), which emphasise the need for specialised education and training in forensic nursing.

### Limitations

Although the study aims to develop the SANE program, it requires widespread acceptance across the hospitals in the country. The absence of policy changes and revision of existing guidelines are a few challenges that can block the nationwide implementation of SANE programs. Sustainable planning is also needed for the development of a platform that connects the SANEs across the country. The study is planned as an experimental design and does not involve any randomisation or control.

## Conclusions

The results from the proposed study are expected to help the nurses working in emergency departments, and outpatient departments and sexual assault referral centres to become knowledgeable, skilful, flexible, non-judgmental, empathetic and understanding, supportive, and resilient. It will also help them to demonstrate adequate coping skills, collaborate, and support other team members. The research will help the nurses to recognise the impact of sexual violence on physical and mental health and provide the necessary care, support and referrals to other requisite services and also add value for the survivor’s medico-legal exam with more sensitivity and accuracy.

## Data availability

### Underlying data

No underlying data are associated with this article.

### Extended data

Figshare: KQSANE.pdf. https://doi.org/10.6084/m9.figshare.18865136.v2 ( Yesodharan *et al*., 2022a).

This project contains the following extended data:

- KQSANE.pdf (The item pool for the development of the tool KQSANE-I)

Data are available under the terms of the Creative Commons Attribution 4.0 International license (CC-BY 4.0).

Fighsare: Informed Consent.doc. https://doi.org/10.6084/m9.figshare.18865130.v3 ( Yesodharan *et al*., 2022b).

Figshare: Participant Information Sheet-Phase 1.doc. https://doi.org/10.6084/m9.figshare.18865139.v2 ( Yesodharan *et al*., 2022c).

Figshare: Participant Information Sheet-Phase 2.doc. https://doi.org/10.6084/m9.figshare.18865133.v2 ( Yesodharan *et al*., 2022d).

Data are available under the terms of the Creative Commons Zero “No rights reserved” data waiver (CC0 1.0 Public domain dedication).
