# Supplementary material for: The effectiveness of a sexual assault nurse examiner-grounding program (SANE-GP) on knowledge, skill and practice regarding sexual assault examination (SAE) among nurses working in a tertiary care hospital in Udupi district, India: A study protocol
Source: F1000Res. 2022 Dec 13;11:134. Originally published 2022 Feb 2. [Version 2] doi: 10.12688/f1000research.74978.2 (PMC9811034; doi:10.12688/f1000research.74978.2)
Supplement: Peer review history

# Peer review: Reviewer response for version 2

**DOI:** 10.5256/f1000research.141999.r158027

**Published:** 2023-01-03

**Authors:** Amita Pitre

**Affiliations:** 1 Lead Specialist, Gender Justice, Oxfam India, New Delhi, Delhi, India; 2 Tata Institute of Social Sciences, Mumbai, Maharashtra, India; 3 Forum for Medical Ethics Society, Mumbai, India

I have seen the revised version of the study protocol. I am approving it for indexing. It will need a copy edit. I also recommend that in all following steps of the study, effort must be made to get 50% women researchers, reviewers etc., and it would be good to have a gender expert/feminist also on board as required. This will make the outcome a more sensitive one.

Is the study design appropriate for the research question?

Yes

Is the rationale for, and objectives of, the study clearly described?

Partly

Are sufficient details of the methods provided to allow replication by others?

Yes

Are the datasets clearly presented in a useable and accessible format?

Partly

Reviewer Expertise:

NA

I confirm that I have read this submission and believe that I have an appropriate level of expertise to confirm that it is of an acceptable scientific standard.

# Peer review: Reviewer response for version 2

**DOI:** 10.5256/f1000research.141999.r158028

**Published:** 2022-12-19

**Authors:** Udara Dilrukshi Senarathne

**Affiliations:** 1 Department of Biochemistry, Faculty of Medical Sciences, University of Sri Jayewardenepura, Nugegoda, Sri Lanka; 2 Monash Health Pathology, Monash Health, Clayton, Vic, Australia

The authors have addressed all the comments given in the initial review.

Is the study design appropriate for the research question?

Yes

Is the rationale for, and objectives of, the study clearly described?

Yes

Are sufficient details of the methods provided to allow replication by others?

Yes

Are the datasets clearly presented in a useable and accessible format?

Not applicable

Reviewer Expertise:

Pathology, Forensic Medicine, Sexual Abuse Examination

I confirm that I have read this submission and believe that I have an appropriate level of expertise to confirm that it is of an acceptable scientific standard.

# Peer review: Reviewer response for version 1

**DOI:** 10.5256/f1000research.78785.r138252

**Published:** 2022-06-28

**Authors:** Amita Pitre

**Affiliations:** 1 Lead Specialist, Gender Justice, Oxfam India, New Delhi, Delhi, India; 2 Tata Institute of Social Sciences, Mumbai, Maharashtra, India; 3 Forum for Medical Ethics Society, Mumbai, India

This appears to be an article based on study design of a prospective study. The rationale of the study needs to specifically mention why they are at all considering the training of nurses given that it is not legal as yet. The rationale also needs to at least flag why SANEs are important.

 I have addressed below what else needs to be included to provide a convincing rationale. Summarising here: 

 Datasets to be used are not completely clear. Only quantitative and statistical data sets seem to have been provided or listed to be used. There will be substantial qualitative information gathered or needs to be gathered from SANE's experiences and if possible those of women; what improved what did not; what are the challenges faced by SANEs; were they given adequate space in a highly patriarchal, hierarchical set up where doctors have the legal privilege to examine. More details below. 

- The literature review can draw more upon the Indian scenario if possible. For example the Henninger citation does not talk about the Indian scenario as far as I can see. So why are Nurses a crucial ally in examination of sexual assault survivors and why they are well placed to be trained as SANEs can be better illustrated from Indian experience if possible. Or then state global evidence shows this and scenario in India is not likely to be different given the important role of nurses in health care provision, including in emergencies.  
- Introduction and Research Gap
  - While providing the rationale of the study and the introduction, you can mention here that law expects a woman to be examiner or given first preference and survivors also find it more acceptable. For gender sensitive care it makes sense to have women examiners. Nurses are almost universally women in India and easier to access than women doctors.
- Inclusion criteria:
  - Here you mention in phase 2 female nurses will be enrolled and earlier in the methodology you mention persons from both genders would be included. It would be good to explain the difference and how and why this has been devised. 
- SANE Grounding Program (SANE-GP); point 2
  - Ideally the spectrum of human genitalia including intersex needs to be added here- not just male and female. Intersex and transpersons also face substantial risk of sexual assault.
- SANE Grounding Program (SANE-GP); point 2
  - Perspective in the entire paper is very medical and may risk objectifying the survivor. E.g. medical management of case instead of provision of health care to survivor. However this should not amount to language dressing but needs to permeate the intent and roll out of study and will then naturally flow in the paper.
- Ethical considerations:
  - This is very inadequate for such a sensitive topic. Please mention specific anticipated ethical concerns such as trauma that potential participants may face, how you will prepare them for it, what care will be available, what will be done to prevent it; several ethical concerns related to mandatory reporting to police- sometimes survivors forego medical treatment to escape reporting - how will that be taken care of; legally nurses are not allowed to practice and in future after the research they may not be allowed to - how will their anticipations be managed; how will the research prevent duplication of examination in case the forensic expert is supervising; how will the survivor be informed about their inclusion in a research- what safeguards, informed consent available to them etc will nurses be able to testify in court etc. These need to be robustly stated and potential resolutions and risks noted.
- Competency of nurses:
  - Strengths of nurses need also to be listed. Why nurses? Women, known to be more sensitive to patients, better at developing rapport with the survivor, would put her at ease, better acceptance of examination, better history taking since she is a woman and easier for women to open up etc. instead of only focusing on building the competency of nurses, state why SANE program would be better than simply training more doctors. 
  - About knowledge of nurses in forensic examination- It has been stated that they do not have the subject in their course and they are not legally allowed to examine. Therefore this statement about their lack of knowledge needs to be qualified with this information. 
  - Instead of only focusing on lacunae in nursing training, need to highlight how training of nurses is also in the interest of women, health system, better delivery of service. As women they will also be able to better empathise and understand the trauma of the woman.
- Methodology 
  - Methodology needs to also include feedback from survivors. It could also have a control group- cohort with SANE involvement and without.
- Discussion
  - The discussion section is very limited and focused on the need to train nurses. However, it needs more depth about the shortcomings of a doctor led model, the critique of the current system, lack of knowledge of doctors themselves and shoddy services despite having it in medical curriculum and specific training, dissonance between doctors actually invited to examine (gynaecologists and women doctors) vs those who are trained (forensics).
  - There is ample literature on current limitations of the medical model and system of examination- most recent by Lakshmi Lingam, Sunita Bandewar and Sita Mammudipudi from Tata Institute of Social Sciences for the ICMR; Human Rights Watch report of a few years back. Multiple writings including my own. All these need to be present from literature review stage onwards and brought together in discussion. So limitations of current model, strengths of nurses doing exam, need for their training and how it may improve the system, legal limitations in doing so, future challenges all need to come together in discussion. 
- Conclusion:
  - Again this is very limiting. The aim is not to enlighten nurses but to add value for the survivors medico-legal exam, more sensitivity, accuracy, etc etc. The heart of the matter is not captured in the discussion, conclusions and the entire approach of the research is to educate nurses. Needs above changes.

Regarding additional literature, here are two studies (one of which I technically reviewed and gave inputs to) and one letter to the editor (I co-authored), which is useful to draw upon how the system for medico-legal examination in India is still poorly developed.

- Enhancing the Quality of Response of the Health Care System to Sexual Assault.
- Dignity on Trial - India’s Need for Sound Standards for Conducting and Interpreting Forensic Examinations of Rape Survivors
- Doctors in India continue to traumatise rape survivors with the two-finger test
- Need for gender sensitive health system responses to violence against women and children

Is the study design appropriate for the research question?

Yes

Is the rationale for, and objectives of, the study clearly described?

Partly

Are sufficient details of the methods provided to allow replication by others?

Yes

Are the datasets clearly presented in a useable and accessible format?

Partly

Reviewer Expertise:

I have a masters in Health Sciences, and extensive work in the response of the health system to sexual assault. I am the Lead Specialist, Gender Justice at Oxfam India. I have done extensive work in gender based violence. I have several publications linked to the same which are accessible on my Academia.edu and Researchgate sites. I was among the first ones to talk about the need for standard protocols and gender training including technical competency training for doctors in examination of sexual assault survivors. I have written extensively on survivor centric approaches to care.

I confirm that I have read this submission and believe that I have an appropriate level of expertise to confirm that it is of an acceptable scientific standard, however I have significant reservations, as outlined above.

## References

[1] : Enhancing the Quality of Response of the Health Care System to Sexual Assault. *Tata Institute of Social Sciences, Forum for Medical Ethics Society* .2021; Reference source

[2] : Dignity on trial India’s Need for Sound Standards for Conducting and Interpreting Forensic Examinations of Rape Survivors. *Human Rights Watch* .2010; Reference source

[3] : Doctors in India continue to traumatise rape survivors with the two-finger test. *Scroll.in* .2017; Reference source

[4] : Need for gender sensitive health system responses to violence against women and children. *Indian Journal of Medical Ethics* .2018; 10.20529/IJME.2018.011 10.20529/IJME.2018.011 29439939

# Peer review: Reviewer response for version 1

**DOI:** 10.5256/f1000research.78785.r135825

**Published:** 2022-05-12

**Authors:** Udara Dilrukshi Senarathne

**Affiliations:** 1 Department of Biochemistry, Faculty of Medical Sciences, University of Sri Jayewardenepura, Nugegoda, Sri Lanka; 2 Monash Health Pathology, Monash Health, Clayton, Vic, Australia

This study protocol addresses an unmet need in the health sector, especially in Asian countries. Although the nurses are pretty involved in the emergency department patient care and ward patient care, they do not routinely receive training on examining a sexual assault victim. Such training is vital as it is essential to obtain useful evidence, administer appropriate treatment measures, educate the victim on certain aspects, and, most importantly, avoid re-traumatizing the victim during the healthcare processes. 

 This study protocol addresses all necessary steps in developing such a training program and auditing its outcome. 

 Since this is the first time such a program would be introduced to the system, I suggest adding a qualitative interview-based section to Phase II in assessing the effectiveness of the SANE-GP by interviewing randomly selected participants using open/close-ended questions to enrich the study outcome.

Is the study design appropriate for the research question?

Yes

Is the rationale for, and objectives of, the study clearly described?

Yes

Are sufficient details of the methods provided to allow replication by others?

Yes

Are the datasets clearly presented in a useable and accessible format?

Not applicable

Reviewer Expertise:

Pathology, Forensic Medicine, Sexual Abuse Examination

I confirm that I have read this submission and believe that I have an appropriate level of expertise to confirm that it is of an acceptable scientific standard.

# Response

**Published:** 2022-05-31

**Authors:** RENJULAL YESODHARAN

**Affiliations:** Manipal University, India

Dear Dr Udara,

 Thank you for reviewing our work.  We will look into the suggestions you have given. A qualitative study will be initiated after completing this project.
